# Reliable reconstruction of cricket song from biophysical models and preserved specimens

**DOI:** 10.1098/rsos.251005

**Published:** 2025-07-30

**Authors:** Ryan Weiner, Sarah Duke, Gabriella Simonelli, Nathan W. Bailey, Natasha Mhatre

**Affiliations:** ^1^ Department of Biology, University of Western Ontario, London, Ontario, Canada; ^2^ School of Biology, University of St Andrews, St Andrews, UK

**Keywords:** bioacoustics, crickets, morphological signal evolution, biophysical modelling, finite element analysis

## Abstract

Predicting the function of a biological structure solely from its morphology can be a very powerful tool in several fields of biology, but especially in evolutionary reconstruction. In the field of invertebrate acoustic communication, reconstructing the acoustic properties of sound-producing forewings in crickets has been based on two very divergent methods, finite element modelling (FEM) and vibrometric measurements from preserved specimens. Both methods, however, make strong simplifying assumptions that have not been tested and the reliability of inferences made from either method remains in question. Here, we rigorously test and refine both reconstruction methods using the well-known *Teleogryllus oceanicus* model system and determine the appropriate conditions required to reconstruct the vibroacoustic behaviour of male forewings. We find that when using FEM it is not necessary to assume simplified boundary conditions if the appropriate parameters are found. When using preserved specimens, we find that the sample needs to be rehydrated for reliable reconstruction; however, it may be possible to accomplish rehydration *in silico* using FEM. Our findings provide a refined methodology for the reliable reconstruction of cricket songs, whether from fossils or preserved specimens from museums or field collections.

## 1. Introduction

Animal acoustic communication signals are extensively studied due to their important role in mediating social interactions such as cooperation, conflict, learning, parental care, sexual selection and reproductive isolation [1,2]. Understanding the causes of acoustic signal variation can be assisted by structure-based reconstruction: in cases where taxa are extinct or unavailable, but archival or fossil specimens remain, reliably reconstructing acoustic signal properties is critical for accurate inferences about signal development, function and evolution. In most vertebrates, vocalizations are based on soft vocal-tract tissues that are under significant neuro-muscular control [3]. In contrast, invertebrate acoustic communication typically relies on vibrations produced by hardened, cuticular structures. For example, crickets produce loud mate attraction calls using specialized hardened forewings [4]; in addition to mate attraction, these calls and their reception by conspecifics function in mate recognition, courtship, sexual selection and aggression [5]. The structural and mechanical properties of hardened cricket wings control most aspects of call structure, such as frequency, duration and even loudness [4,6,7]. Thus, developmental or evolutionary processes that alter wing structure can drive signal evolution, and hard tissues if preserved can carry a record of signal evolution amenable to reconstruction [8,9]. Refining methods of structure-based signal reconstruction from preserved specimens would therefore accelerate our understanding of signal evolution in cricket species [10–12].

An area of particular interest is the effect of wing venation patterns on cricket song frequency. Wing veins are used to delineate the harp and mirrors, which act as resonators in song production [13]. A modified vein, the file, is part of the stridulatory apparatus that excites these resonances using a highly regulated clockwork mechanism [7]. There is now considerable evidence that changes in wing venation patterns are genetically driven, and that these changes have been a strong driver of observable signal evolution in crickets [10–12,14]. Some researchers have argued that vibrational patterns from preserved specimens can be used to infer the patterns of vibrations in live crickets because venation is the structural feature that is critical [15]; however, there is no direct empirical or modelling evidence showing that this is the case.

Most biomechanical models that predict cricket wing resonances from structure typically use the finite-element (FE) method [9,16–18] and make strong simplifying assumptions about how veins provide the boundary conditions of the system. Current models assume that all the external boundaries of the resonating wing are clamped or held immobile. They justify this assumption by arguing that in regions of the wing with a high density of veins, we typically observe high stiffness and low movement levels in vibrometric measurements, and it is therefore reasonable to consider these boundaries to be immobile or ‘clamped’ [16,17]. In some cases, an additional boundary condition has been considered. During singing, when the file and plectrum engage to produce the forces that cause the wing to vibrate have also been treated as a contact boundary condition by previous authors [16,19,20]. However, in real wings, as they are typically tested vibrometrically to excite the song resonance, only the base of the wing is truly clamped and other wing edges are free to move [13]. It has not been tested whether FE models with these vibrometrically relevant boundary conditions capture the reduction in vibration observed in high vein density areas. Indeed, we do not have a systematic way of deciding what vein density would render a part of a wing become immobile and for what frequency range.

For instance, in *Teleogryllus oceanicus* from Hawaiian populations, a mutation called *flatwing* silences males and is maintained by selection pressure from the acoustically orienting parasitoid fly *Ormia ochracea* [14]. Silent *flatwing* males have partially feminised forewings with very high density of veins throughout the wing and a very reduced harp [10]. Therefore, if we used the boundary condition proposed by existing FE models, we would have to assume that all regions of the *flatwing* wing are stiffened and immobile, a circular statement without any need for an FE model. Additionally, this model class would have little to say about the newer wing variants observed in *T. oceanicus* which have a range of vein densities and wing sizes [14,21]. Indeed, some of these wing variants have reduced but functional singing structures like the file and plectrum [21] and others possess vestigial movements associated with singing [22], which may give rise to novel forms of sound production that would be missed by current methods. Thus, we cannot use the simplifying assumptions currently used in FE models of cricket wings for realistic structural reconstruction of wing resonances across the substantial diversity of forewing venation patterns and densities found both within and across cricket species. To accurately reconstruct the transition from non-singing to singing species, there is a considerable need for models that explicitly model these stiffening structures and their effects on wing resonances and vibration.

While it might be the case that stiffening structures such as veins determine the spatial pattern of cricket wing vibrations, it is not clear that they determine the frequency of this vibration as has been assumed in studies of preserved specimens, another method used for the reconstruction of acoustic function [15]. It is well known that insect cuticle becomes stiffer when desiccated, as captured by an increase in the Young’s modulus, and also that it experiences reduced damping [23,24]. Increases in wing cuticle stiffness will also increase the stiffening effects of the veins since they are made of the same material. Thus, resonances measured from preserved specimens are expected to be at higher frequencies than those from live animals. Indeed, resonance frequencies cannot be predicted from morphological features alone; the Young’s modulus of insect cuticle varies considerably [25], and so do simple morphological features like wing cuticle thickness, both of which affect resonance frequency [18].

To fill this gap in our understanding of how cricket wing resonances are determined by their structure, we developed a FE model of *T. oceanicus* wings where we used realistic boundary conditions, clamping only the wing base and explicitly modelling the stiffening effect of wing veins using a coupled plate and rod formulation. We began by developing a model of wildtype (‘normal-wing’, Nw) males using known morphology and measurements of wing vein diameters (electronic supplementary material, figure S1). We then used a process of fitting and iterative refinement to infer two unknown mechanical properties that are responsible for the resonance frequency, i.e. the Young’s modulus of wing cuticle (*E*) and the thickness of the wing membrane (*mt*) (see electronic supplementary material for details, figure S3). Next, we used further refinement to estimate the damping in the system. We used these mechanical properties and the known morphology of *flatwing* (Fw) mutant wings to test if a model of these wings predicts the loss of resonance observed in the real insects [10].

Additionally, we conducted a second test of our models and test whether we can also predict the resonance behaviour of dry-preserved wing specimens and tested their relationship to live specimens. Dry-preserved insect cuticle is known to become stiffer, with an elevated Young’s modulus, and experience lowered damping [23,24,26]. These parameters can be independently modified in our model and would allow us to estimate accurate values for the resonances of preserved wing specimens and their relationship to live wings. Thus, using a combination of FE modelling and vibrometry, we can identify which features of the singing structures of cricket wings can be reliably inferred from preserved or fossil specimens for the reconstruction of signals and evolutionary analysis.

## 2. Methods

### 2.1. Finite element model construction

All FE models were developed in COMSOL Multiphysics (v. 5.5 and 6.1; Burlington, MA, USA). Detailed methods are provided in the electronic supplementary materials. Vector drawings of *T. oceanicus* wings and venation patterns were imported into COMSOL to provide model geometry. We ran two wing models (electronic supplementary material, figure S3). In the first model, the entire wing was in a single plane, i.e. a flattened wing. In the second model, the folded wing model, the lateral field was folded at a 45° angle, as it naturally would be when the animal was singing. In all models, the wing veins were defined as beams completely coupled along their length to the wing membrane, which was defined as a plate with defined thickness. Vein thicknesses were measured using optical coherence tomography (OCT; electronic supplementary material, figure S2), and mean values were used with a sensitivity analysis for harp vein diameter (electronic supplementary material, figure S5 for detailed methods). Wing membrane thickness was below OCT resolution (4 µm) setting an upper bound for the possible thicknesses at a maximum of approximately 8 µm. The best fit parameter for this thickness was found through iterative refinement, along with the Young’s modulus of the wing and vein cuticle. For damping, we initiated the model with Rayleigh damping parameters derived from vibrometry measurements as described before [16] and then iteratively refined damping estimates using the isotropic loss factor formulation. Typically, insect wing veins are a mix of solid structural veins and hollow veins that carry blood and axons from sensory neurons, which are distributed across the wing [27,28]. In the models reported here all veins were treated as solid rods, since this replicated real behaviour. When the veins were treated as hollow pipes with a wall thickness set at the same level as membrane thickness, the models did not replicate wing behaviour suggesting that in cricket wings modified for singing, veins are predominantly solid and structural in nature.

First, we conducted a series of eigenfrequency studies in COMSOL for all parameter combinations under consideration (electronic supplementary material, figure S3). An eigenfrequency study finds the resonance frequencies of the modelled system and the corresponding spatial vibrational patterns or modes at each eigenfrequency. Then we identified the subset of these parameter combinations that were close to model fit criteria.

**Figure 1.**
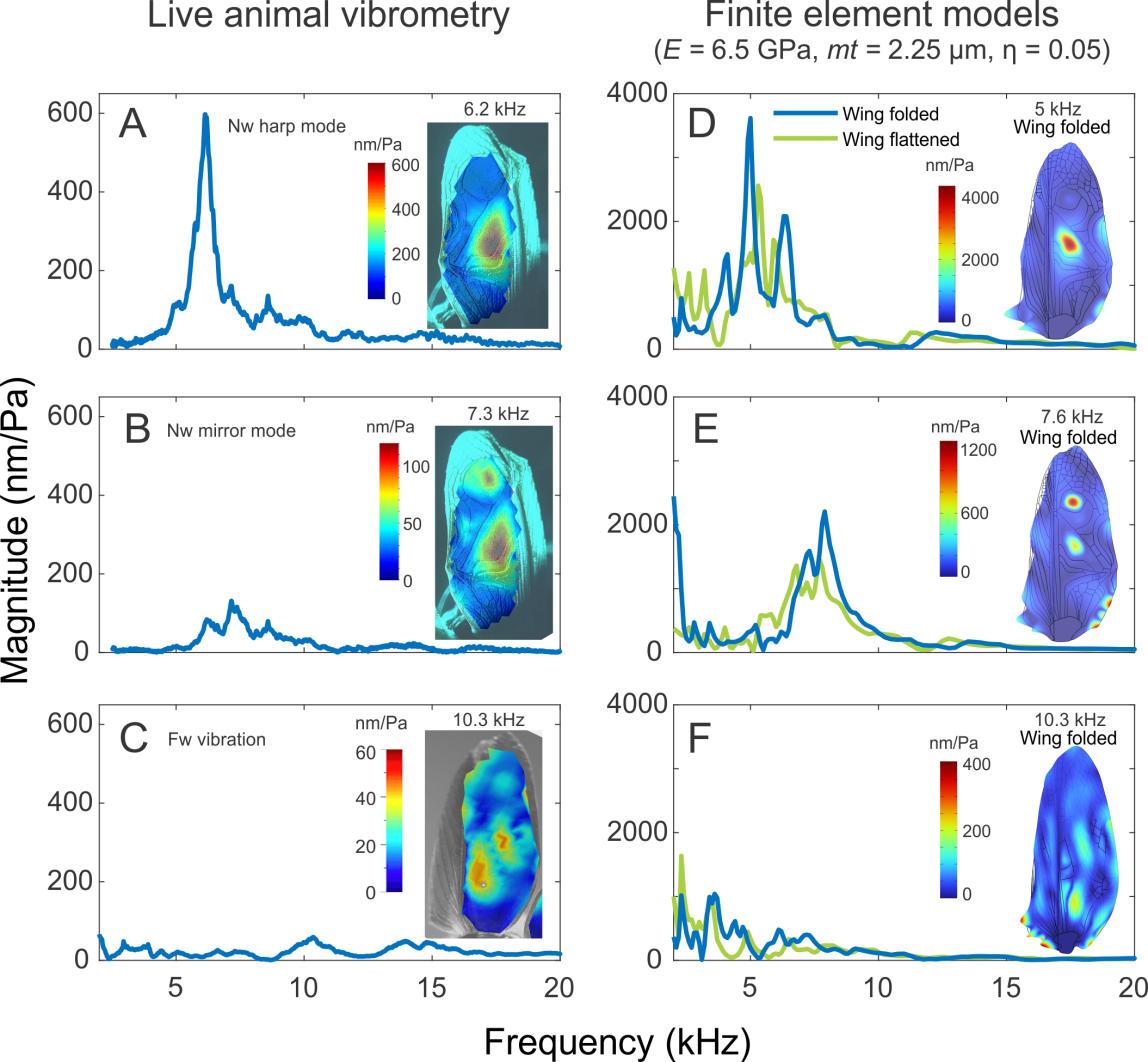
Finite element models capture the vibrational patterns of real *Teleogryllus oceanicus* wings. The singing forewings of *wildtype* or normal winged *T. oceanicus* crickets (A) have a sharp peak in their frequency response near their singing frequency which corresponds to a resonant mode which we refer to as harp mode or mode 1. To observe this peak in the frequency response, we plot the frequency response at a central point on the harp which is depicted in the figure. (B) Their forewings have another resonant mode at a higher frequency where the harp vibrates out of phase with the mirror cells. To observe the appropriate frequency response for this mode, we plot the frequency response of a central point on the mirrors which is depicted in the image. (C) In *T. oceanicus* crickets that carry one of the *flatwing* mutations, the forewing has an altered venation structure, an altered frequency response with significantly lower vibration amplitudes. A finite element model that incorporated the appropriate geometry of the normal *T. oceanicus* wing including a folded lateral field and had a realistic parameter set for the wing cuticle modulus and thickness showed (D) a harp mode at a song like frequency and had a very similar frequency response from the same point on the harp in terms of both the resonant frequency and the Q factor (see results). The same model also reproduced (E) mode 2 in which the harp and mirror show anti-phase vibrations. The frequency response of the modeled mirror resembled that of the real mirror in terms of both the resonant frequency and frequency response (see results). When the same model parameters were applied to the wings of flatwing mutants, the model predicted vibration shapes that were very similar to those observed in real wing and which had very similar low amplitude frequency responses. The frequency responses shown in panels A, B and C represent single individuals.

Model criteria were based on the mechanics of live wildtype *T. oceanicus* wings. Data for wildtype wings were taken from previous work (two animals) [10], and further data were collected for this study (four animals, see electronic supplementary material for details). Model criteria included (i) the prediction of a resonant mode in which only the harp vibrated between 4.8 and 6.2 kHz with a Q factor between 5 and 13 and (ii) a second mode between 6 and 8.8 kHz where the mirrors also showed significant anti-phase vibrations, which had a magnitude at least 2 times lower than the first resonant mode (see electronic supplementary methods for details). Only resonance frequencies and mode shapes can be identified exactly in eigenfrequency studies. Therefore, only these parts of the criteria could be evaluated in these studies. For the subset of parameter combinations that partially met model criteria, we conducted frequency domain studies that are more computationally expensive but generate estimates of the Q factors and also the relative amplitudes of different modes. In these frequency domain studies, we examined the vibrational behaviour of the wings in response to a constant pressure (20 mPa or 60 dB SPL re. 2 × 10^−5^ N m^−2^) applied to the wing across a range of frequencies (2–25 kHz). Outputs from this study were then used to examine the frequency response of different points on the wing (figure 1).

### 2.2. Vibrometry of preserved wing specimens

In 2019, forewings were collected from normal-wing and flatwing males obtained from laboratory *T. oceanicus* lines. The lines were pure-breeding for the respective genetic variants and were originally derived from a population in Kauai, Hawaii, where the two morphotypes segregated at the time of collection [29]. After removal with dissecting scissors, forewings were mounted on cardboard with a small piece of Blu Tack (Bostik), placed in an insulated tube and maintained dry until use approximately 5 years later. Dry-preserved wings were rehydrated as needed by submerging into distilled water for about 1 h directly before measurement. To facilitate comparisons, we used laser Doppler vibrometry to measure the vibrational behaviour of both dry and rehydrated forewings in response to acoustic stimulation at a range of frequencies similar to those used for live animals [10]. These data were used to identify the harp mode, and its modal frequency, amplitude and the Q factor of the modal frequency response (see electronic supplementary materials for detailed methods). All three metrics were compared between dry and rehydrated wings using paired *t*-tests in R (RStudio v. 1.4.1717). Similarly, all three metrics data were also compared with vibrometry data from live *T. oceanicus* wings using unpaired *t*-tests. Errors for the foregoing tests were symmetrically distributed. Data from the forewings of live animals were used as a baseline to evaluate the effects of preservation and subsequent rehydration.

## 3. Results

### 3.1. Models of normal wings

We used data from vibrometric measurements of the wings of living wildtype individuals to assess whether the model output fit real wing behaviour (electronic supplementary methods for details). In the first iteration of eigenfrequency studies for the Nw model (see electronic supplementary methods, figure S3), we tested a broad range of parameters (Young’s modulus (*E*) = 1–10 GPa and membrane thickness (*mt*) = 2–10 μm) at a low resolution (1 GPa and 1 μm, respectively). These model parameter ranges were based on previously reported data from other cricket species [18]. The model was first set to output eigenfrequencies between 4 and 6 kHz, and the resulting mode shapes for each parameter combination. We examined these eigenfrequencies to see if they possessed a mode that was isolated to the harp with an anti-node centred in the harp which fell within the natural range, i.e. between 4.8 and 6.2 kHz.

Next, we ran a second iteration of eigenfrequency studies (electronic supplementary methods, figure S3) using a narrower range of parameters (*E* = 5–10 GPa and *mt* = 1–5 μm) surrounding the few that fulfilled the first criteria. These were run at the same resolution as the previous iteration (1 GPa and 1 μm, respectively); however, the eigenfrequency search was now extended and went from 4 to 10 kHz. Within this range, we checked whether the parameter combinations also produced a second mode with antiphase movement in the mirrors. We found that the *E* values that would reproduce the main harp modes between 4.8 and 6.2 kHz and showed some movement in the mirrors at higher frequencies were between *E* = 6 and 7 GPa and when membrane thickness was either 2 or below 3 μm. Eigenfrequencies do not give us a clear indication of the full frequency response and therefore of the Q factor of the resonances. They also do not predict which modes are superposed on each other when driven at a particular frequency. Therefore, further parameter refinement was achieved through a frequency domain study.

To identify which parameter combination best captures Nw wing resonance, we conducted a frequency domain study searching for *E* = 6–7 GPa and *mt* = 2–2.5 μm, at a higher resolution of 0.5 GPa and 0.25 μm, respectively (see electronic supplementary methods, figure S3). The frequency responses for a point on the harp and the mirror-cells for each combination of *E* and mt were analysed to see which parameter combination best met our criteria. The first part of our criteria was that the harp’s frequency response should show a single peak frequency between 4.8 and 6.2 kHz (5.61 ± 0.53 kHz, mean ± s.d., *n* = 6 real animals) with a Q-factor between 5.5 and 13 (7.9 ± 2.77, mean ± s.d., range 5.5−13.1, *n* = 6 animals). We also set the criteria that the mirror mode or the second mode in which the mirrors move in an antiphase fashion to the main harp will occur at a frequency approximately 1.3 times higher, and between 6 and 8.8 kHz (7.2 ± 0.92, peak frequency ratio: 1.3 ± 0.13, *n* = 6 animals) than the harp but at least 2.0 times lower in magnitude (harp mode: 413.67 ± 132.86 nm Pa^−1^, mean ± s.d.; mirror mode: 119.57 ± 58.91 nm Pa^−1^, mean ± s.d.; peak height ratio: 4.45 ± 3.3, mean ± s.d., range: 2.0–10.1, *n* = 6 animals). The mirror mode is highly variable in real animals and is not always the peak frequency of the mirrors; hence, we consider this a weak criterion.

Additionally, we do not expect exact matches in displacement levels at either mode from the models and do not use these in our model fit criteria. In the vibrometry measurements, the wings are stimulated by a sound that will apply force on both faces on the wing suspended in the sound field; therefore, the exact net force level on the wings is unknown. In the model, we apply pressure only in one direction, thus generating a higher force on the wings. Thus, the force levels and therefore the absolute displacements between the model and real wings cannot be directly compared. We do, however, expect that the relative displacements between the harp and the mirror mode will be similar between the model and the real wings. Another aspect we consider here is the position of the lateral fold in cricket forewings. The wings of crickets have a lateral field that is usually held at an angle to the plane of the main wing when singing. This fold occurs naturally when wings are raised, including when wings are measured using a vibrometer. Recent work in plate mechanics has shown that such structural folds and bends have the capacity to sharpen resonances, focus modes and stiffen plates [30]. To capture the variation in mechanical behaviour introduced by the positioning of the lateral field, here we report models with a flattened and folded lateral field.

The best combination of parameters was found to be *E* = 6.5 GPa and *mt* = 2.25 μm (figure 1) which are congruent with data from other cricket species [18]. Initial values of damping used were then updated, and we found that an isotropic loss factor (*η*) of 0.05 gave us the best fit between models and real animals. In the model where the lateral field was flattened, at the harp, the highest peak frequency occurred at 5.3 kHz with a magnitude of 2562 nm Pa^−1^. When the lateral field was folded, the peak frequency shifted slightly lower to 5 kHz and had a magnitude of 3615 nm Pa^−1^. The shape of the vibrational mode predicted by the model was similar to that seen in the real data, with a single high amplitude anti-node present in the centre of the harp with smaller associated movement in the mirrors (figure 1; electronic supplementary material, figure S4). In the flattened wing, we could see some movement along the vertical veins (M, Sc and R, figure S1), which demarcate the lateral field (electronic supplementary material, figure S4). This movement is reduced in the folded model, and movement of the harp spreads a bit towards the three harp veins (figure 1). The Q factor of the harp vibrational mode was approximately 5.88 in the flattened wing model and 12.5 in the folded wing model, both within ranges observed in real measurements. However, it is clear that folding the wing significantly enhances wing resonances, a mechanism likely to be exploited by crickets during singing (figure 1). We also found that varying the diameters of the three harp veins (Int I–III, electronic supplementary material, figure S1) did not significantly alter the resonance frequency (electronic supplementary material, figure S5). Wings with lower diameter veins had a sharpened harp mode resonance, and the modal shape spread lower through the three internal harp veins (electronic supplementary material, figure S5).

At the mirror-cells, there was a peak frequency centred around 7.6 kHz for both models with a magnitude of 1097 nm Pa^−1^ for the flattened wing model and 1237 nm Pa^−1^ for the folded wing model. In the flattened wing model, this is at a frequency that is 1.4 times the harp resonance frequency and in the folded wing model, this is at 1.5 times the harp resonance frequency. The magnitude of the mirror resonance is 2.3 times lower that the harp’s resonance in the flattened wing model and 2.9 times lower in the folded wing model. The shape of this vibrational mode predicted by the model was also similar to that seen in the real data, with two out-of-phase anti-nodes in the harp and the mirror, respectively. Thus, in our models the parameter combination of *E* = 6.5 GPa, *mt* = 2.25 μm and an isotropic loss factor (*η*) of 0.05 provided the best prediction of the resonance of *T. oceanicus* normal wings in terms of capturing wing resonant frequencies and modal shapes with the folded and flattened model encompassing the natural variability we observe in laser Doppler vibrometry (LDV) measurements.

### 3.2. Models of *flatwing* mutants

Using this parameter combination, we modelled the flatwing *T. oceanicus* mutant where the wing itself was modelled both ways such that the lateral field was either flattened or folded. In both cases, the frequency response for a point at the vestigial harp in the Fw rod model displays numerous very small peak frequencies and a large overall reduction in movement that is comparable to the observed reduction in forewing vibration amplitude in comparison to Nw males (figure 1) as reported in a previous study [10]. In the real data, we see some displacement localized to the vestigial harp at 10.3 kHz, with similar movement levels at other positions on the wing. Both models also show similar movement at the vestigial harp and across the wing at 10.3 kHz. Thus, our Fw FE model also matches the mechanical behaviour of the real Fw, indicating that venation pattern is primarily responsible for determining the spatial pattern of wing resonance in *T. oceanicus* wings.

### 3.3. Measurements and models of preserved wing specimens

**Figure 2.**
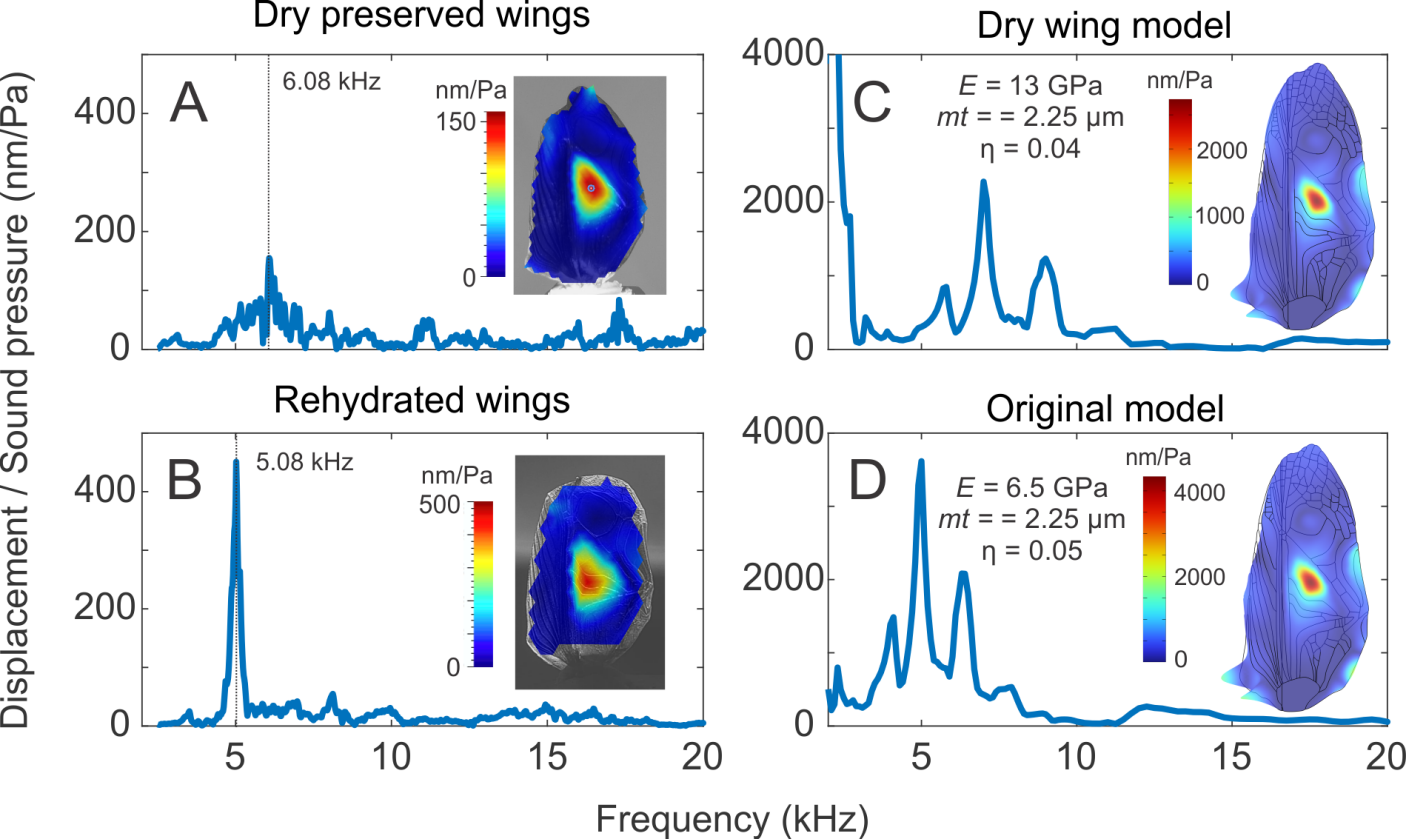
Dry-preserved forewing specimens can be informative about the vibrational mechanics of live animals. (A) LDV measurements from dehydrated dry-preserved *Teleogryllus oceanicus* wings have a harp mode. However, this mode occurs at a higher frequency than one observed in live animals as can be observed from the frequency response at the harp. (B) When rehydrated, the harp mode is retained, and the resonance frequency decreases as can be observed from the frequency response at the harp and is the same as that observed from live animals. (C) A finite element model with a folded lateral field and a Young’s modulus that is twice that of the (D) live normal wing model captures the frequency response observed at the harp of dehydrated dry-preserved wing, coupled with a small decrease in damping. This is consistent with previous observations that the Young’s modulus of desiccated insect cuticle is known to increase, and its damping is known to decrease [23,24,26]. Thus, both data and models suggest that models and dry-preserved wing specimens could be used to reconstruct the vibrational mechanics of cricket forewings following careful analyses.

We then measured the vibrational behaviour of *dehydrated* dry-preserved specimens of Nw *T. oceanicus* collected from the Kauai lineage that had been dry preserved since 2019. We found that that when excited by sound, these wings had a harp mode that appeared very similar to that of live Nw *T. oceanicus* wings (figure 2). The harp mode in these desiccated wings occurred at a significantly higher frequency (6.76 ± 0.84 kHz, *n* = 10) than that observed in live specimens (5.61 ± 0.53 kHz, *n* = 6, 1-sided unpaired *t*‐test, *t*-stat = 3.34, *p* = 0.0024). It is well known that the Young’s modulus of insect cuticle increases when excised and dehydrated [23,24,26]; thus, this is not an unexpected result. We additionally observed a decline in displacement levels in dry-preserved wings (230.68 ± 52.91 nm Pa^−1^, *n* = 10) in comparison with live wings (413.67 ± 132.86 nm Pa^−1^, *n* = 6, 1 sided unpaired *t*‐test, *t*-stat = −3.22, *p* = 0.009) consistent with an increase in stiffness. The wing modes also had a higher Q factor in dry-preserved wings (51.04 ± 24.63, *n* = 10) than live wings (7.98 ± 2.77, *n* = 6, 1 sided unpaired *t*‐test, *t*-stat = 5.47, *p* = 0.00017) suggesting an expected decline in material damping; however, the Q factor in the preserved wings was harder to estimate due to the ‘rough’ nature of the frequency response. These data are consistent with a model with Young’s modulus that has increased by a factor of two to 13 GPa, and a lower isotropic loss factor (η) of approximately 0.04 (figure 2).

**Figure 3.**
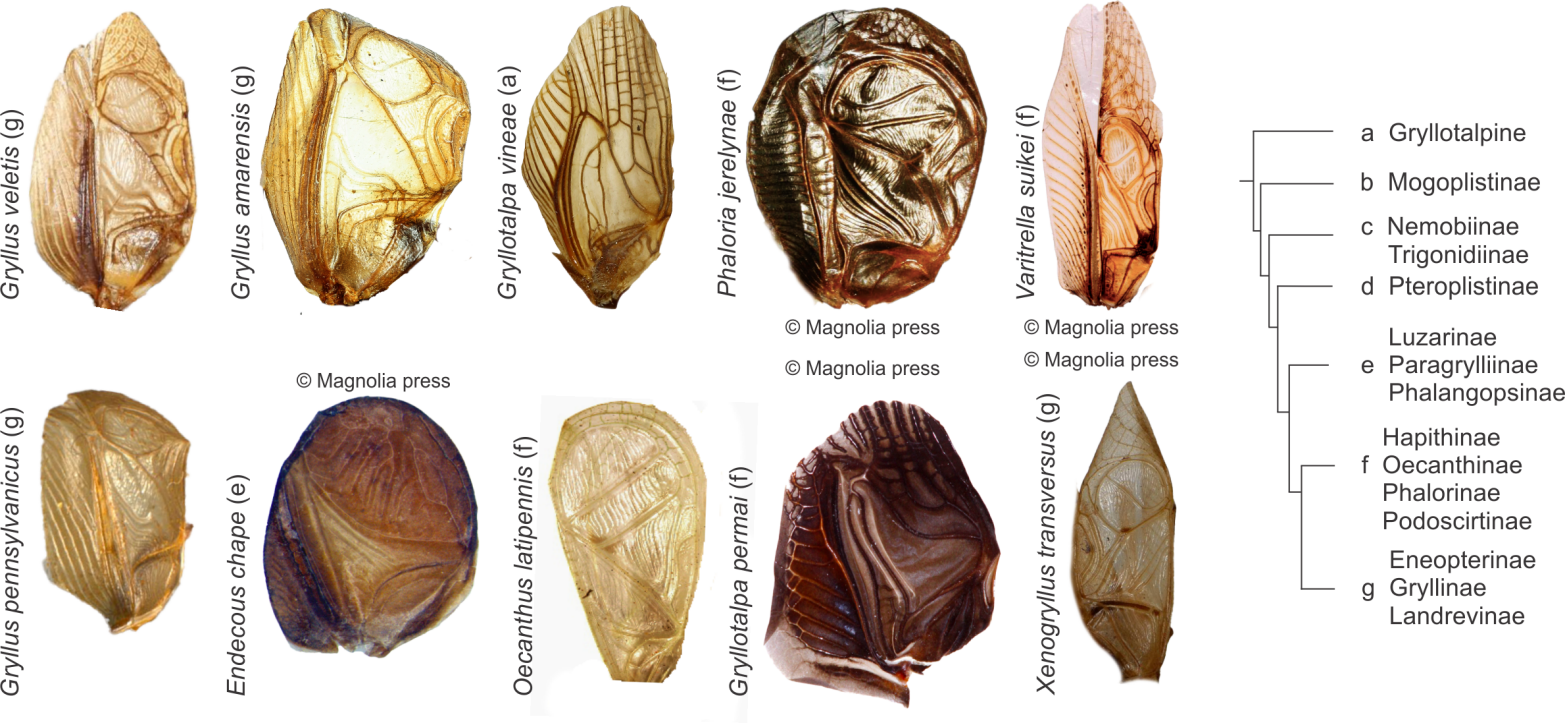
A few example cricket forewings from acoustically active members of the true crickets (Grylloidea) depicting the diversity of wing shape, venation and sclerotization observed across the phylogeny. Images of the following species are reproduced under a CCBY license, and specimen details are provided in the electronic supplementary material: *Gryllotalpa vinae* (by Marion Depraetere), *Gryllus veletis* (by Holger Braun), *Gryllus pennsylvanicus* (by Don Griffiths), *Gryllus amarensis* (by Ranjana Jaiswara) and *Oecanthus latipennis* (by CBG photography group). Images of the following species are reproduced with permission from the copyright holder from sources that are cited after each species name. Further details are provided in the electronic supplementary materials: *Endecous chape* [32], *Phaloria jerelynae* [33], *Gryllotalpa permai* [34], *Varitrella suikei* [35] and *Xenogryllus transversus* [36]. The *G. pennsylvanicus* photo is courtesy of the Spencer Entomological Collection, Beaty Biodiversity Museum, UBC.

The Young’s modulus of dessicated insect cuticle is also known to return to live values upon rehydration [23,24]. When rehydrated for a period of approximately 1 h, the frequency of the harp mode (figure 2B) lowered to frequencies (5.08 ± 0.25 kHz, *n* = 10) similar to those of live crickets (5.61 ± 0.53 kHz, *n* = 6, 1 sided unpaired *t*‐test, *t*-stat = −2.33, *p* = 0.97). The displacement levels of the rehydrated wings remained lower and were more variable (175.12 ± 133 nm Pa^−1^, *n* = 10, 1 sided unpaired *t*‐test, *t*-stat = −3.47, *p* = 0.002), and the mean Q factor also remained elevated (16.55 ± 7.78, *n* = 10, 1 sided unpaired *t*‐test, *t*-stat = 3.18, *p* = 0.0038), suggesting that material damping remains high even after rehydration.

## 4. Discussion

### 4.1. Venation and spatial pattern of vibration

Our data show that FE models based on the rod formulation can capture the mechanics of cricket forewing resonances, without additional constraints or strong boundary condition assumptions. We quantified the fit of our models by testing four parameters of the harp mode: modal shape, modal frequency, Q factor and modal amplitude relative to mode 2. We also quantified the fit of the model by testing similar parameters for the second resonant mode, the mirror mode: modal shape, modal frequency and modal amplitude relative to mode 1. The model fits on these six highly quantitative and independently varying criteria. Additionally, we find that comparisons between the vibration patterns observed in *T. oceanicus* normal wings and *flatwing* mutant models were predictable based on our models.

Where no other parameter was changed, our models support the idea that high vein densities can reduce vibration locally and suppress the formation of modes. However, antinodes are formed in the harp and also in the mirrors at higher frequencies, despite the presence of intersecting veins in both structures (figure 1). Thus, our models show that while it is not possible to *a priori* predict the vein density required to significantly suppress local vibration*,* it is possible to predict vibration patterns by explicitly modelling real veins and wings with realistic boundary conditions.

One relevant limitation of our analyses is that we assumed that all veins are either rod- or pipe-like in nature, whereas in reality a wing may have some combination of both. We also make the assumption that the wing is of uniform thickness, whereas there may be local variations in cuticle thickness. Nonetheless, our data suggest that wing venation and its stiffening effects are the most critical to forming the appropriate spatial patterns of resonant excitation, known as resonant modes.

The harp mode is critical because the harp is bordered by the file vein, and this vein must vibrate in order to enable the clockwork mechanism that couples and maintains the plectrum drive at the resonant frequency [4,7,13]. Thus, making a reliable prediction of the shape of the harp mode or any mode that would excite the file vein is important since it predicts which frequencies could shape the clockwork mechanism. For instance, both harp modes and higher frequency vibrational modes of forewings might be important to the production of calling song, as has been speculated in some eneopterines [31] and observed in tree crickets [16]. In tree crickets, the spatial pattern of these higher modes can be seen to involve the file vein, explaining the ability of the tree cricket clockwork mechanism to work at a broader frequency range [16].

In *T. oceanicus*, both the harp mode and mirror modal shapes remained similar with some variations in the spread of the antinode in real wings in live animals and preserved specimens (figures 1 and 2). In some individuals, the harp mode vibration remains confined to the upper part of the wing, and in others, it spreads to include the harps internal veins. Models predict similar modes across a wide range of cuticle stiffness; however, a great deal of the variation in the spread of the mode has been captured by our sensitivity analyses, whether in macro-level variations in geometry such as the folding of the lateral field (figure 1; electronic supplementary material, figures, S3 and S4) or micro-level variations in vein diameter (electronic supplementary material, figure S5). Incorporating further variations in cuticle stiffness, thickness or vein type especially systematic variations would capture any remaining variation in mode shape, however, would require more detailed data.

That said the relative insensitivity of the model to cuticle stiffness is intriguing since it suggests that variations in stiffness caused either by cuticle sclerotization during development or due to hydration status would not significantly alter the spatial pattern of vibrations across individual crickets. It also suggests that spatial patterns of vibration are possible to infer from desiccated specimens, at least where these modal patterns are the lower frequency high-amplitude modes of the forewings such as the harp. We found that the harp mode of *T. oceanicus* wings was present in both stiffer models and dry-preserved forewings (figure 2), whereas the mirror modes that are at higher frequencies were more variable in the dry-preserved forewings. Thus, where higher frequency vibrational modes of forewings might be important to the production of calling song as has been speculated in some eneopterines [31], the use of dry-preserved or even rehydrated specimens may need to be tested more carefully.

### 4.2. Reliability of inferences from preserved specimens

Vibrometry measurements from dry-preserved specimens of *T. oceanicus* wings, which are expected to have increased cuticle stiffness due to dessication, were indeed found to have increased resonant frequencies but retained a harp mode (figure 2). This is congruent with our models, which also predict that increases in wing cuticle modulus cause increases in resonant frequency but maintain the harp mode shape (figure 2). The frequency responses measured from both the model and the dry-preserved specimens were significantly noisier than those from fresh wings, which is likely caused by the lower damping observed in dehydrated insect cuticle [23,24]. Thus, not the just the frequency but also the shape of the ‘resonance filter’ of cricket wings is altered by dry preservation. Thus, while it is possible to make some inferences about the spatial patterns of vibration in a dry-preserved cricket wing specimen, it is not possible to infer the shape or the frequency of the resonant filter underlying the cricket’s song.

Interestingly, however, we found that rehydration lowered the resonant frequency of dry-preserved wings so that it was the same as that observed in live animals (figure 2). Previous work has shown that the increased Young’s modulus in dehydrated cuticle can be restored to its normal value simply by rehydration [23,24]. Indeed, our data show that rehydrated wings have vibrational mechanics that are statistically indistinguishable from those measured from live animals, where the harp mode is concerned.

This raises the interesting possibility that dry-preserved specimens may be used for investigating wing mechanics, with the caveat that the effect of dehydration and rehydration should be tested for a wider range of cricket wing morphologies (figure 3). A range of possibilities open up if this proves to be the case, such as systematic studies of cricket song biomechanics without the need for live specimens. As an example, it would be possible to examine the mechanics of dry-preserved museum specimens, where paratypes are available to be manipulated via rehydration rather than risking critical type specimens. It would also be possible to study the wing mechanics of new species as they are found without the need for live specimen transfers. Such mechanical studies would further taxonomic characterization in addition to song and could be used to track natural variation and incipient speciation. An additional possibility is to examine mutational variations observed in natural populations [37] or induced in experimental genetic studies, also without the need for shipping live specimens across national boundaries. There will remain some specimens, which are either too fragile or damaged for such an approach, or indeed some fossil specimens where only two-dimensional impressions are available. In such cases, existing approaches [8], which rely on features of the file such as length and teeth number which are known to correlate with song frequency at least in tettigoniids [38], would also be useful in model generation and refinement.

### 4.3. Modelling cricket wing diversity and song evolution

It is worth reiterating at this point that there is a huge diversity of cricket wing structures across the true cricket phylogeny (figure 3). However, even among the gryllines, the relative shapes and sizes of the harp and the mirrors can vary greatly as can be observed in the three members of this genus *Gryllus*, *G. veletis*, *G. pennsylvanicus* and *G. amarensis* (figure 3). Indeed across the gryllid phylogeny, members of which all sing in a similar fashion to gryllines, we see a huge variation in parameters that are likely to affect wing mechanics: wing size, shape, sclerotization and wing membrane thickness (figure 3) [6,18,39]. In particular, there is a huge variation in the venation pattern that different species use to stiffen their wings and therefore to determine wing resonance. In contrast, the diversity of true cricket (Gryllidae) wing mechanics that have been studied so far is relatively small, there are some measurements of wing mechanics of gryllines [10,13,40], oecanthines [16] and some eneopterines [41]. In previous attempts to model singing cricket forewings, researchers have approximated boundary conditions in different ways. Some researchers have used clamped boundaries around the harp, which is considered the ‘main’ resonator in gryllines [9,17], others have treated the edges of the dorsal field which include the harp and mirrors as clamped allowing higher modes to be captured as well [16]. However, both these approaches are approximations and introduce user bias, since in reality the wing is only clamped at the base. Our results show that such approximations are not necessary, and it is possible to recreate quantifiably realistic wing mechanics using realistic boundary conditions when the appropriate rod and plate formulation is used to capture the stiffening effect of the wing venation pattern.

Our study that explores how venation pattern determines vibration mechanics and cricket song therefore opens up the possibility of a model-based approach to studying cricket song diversity. One can quite easily imagine the use of transformation grids, such as those used by D’Arcy W. Thompson [42] or more modern landmarking techniques [43,44] to describe the variations observed in cricket wing venation diversity across the phylogeny in existing databases [6]. Such transformation grids could be used to develop FE models. Existing information about the song frequency of different species [6] could then be used to tune models allowing us to infer Young’s modulus and cuticle thickness for a range of species. The predictions of selected models could be tested, especially those of unusual morphologies or very distinct wing types such as those of oecanthines. These measurements could be made with live and with dry-preserved specimens to further test the utility of our findings here. The results of such a large-scale analysis would enable us to disentangle the interactions between the main morphological determinants of song frequency in the gryllid phylogeny, whether development has favoured wing modulus, size, shape or membrane thickness as the main lever for signal divergence.

## Data Availability

A complete dataset is provided at the following Dryad link [45]. Supplementary material is available online [46].

## References

[1] Bradbury JW, Vehrencamp SL. 2011 Principles of animal communication, 2nd edn. Sunderland, MA: Sinauer Associates.

[2] Ritchie MG. 2007 Sexual selection and speciation. Annu. Rev. Ecol. Evol. Syst. **38**, 79–102. (10.1146/annurev.ecolsys.38.091206.095733)

[3] Suthers RA, Fitch WT, Fay RR, Popper AN (eds). 2016 Vertebrate sound production and acoustic communication. Cham, Switzerland: Springer International Publishing. (10.1007/978-3-319-27721-9)

[4] Bennet-Clark HC. 1999 Resonators in insect sound production: how insects produce loud pure-tone songs. J. Exp. Biol. **202**, 3347–3357. (10.1242/jeb.202.23.3347) 10562517

[5] Alexander RD. 1962 Evolutionary change in cricket acoustical communication. Evolution **16**, 443–467. (10.1111/j.1558-5646.1962.tb03236.x)

[6] Brandt EE, Duke S, Wang H, Mhatre N. 2023 The ground offers acoustic efficiency gains for crickets and other calling animals. Proc. Natl Acad. Sci. USA **120**, e2302814120. (10.1073/pnas.2302814120) 37934821 PMC10655215

[7] Elliott CJH, Koch UT. 1985 The clockwork cricket. Naturwissenschaften **72**, 150–153. (10.1007/BF00490404)

[8] Gu JJ, Montealegre-Z F, Robert D, Engel MS, Qiao GX, Ren D. 2012 Wing stridulation in a Jurassic katydid (Insecta, Orthoptera) produced low-pitched musical calls to attract females. Proc. Natl Acad. Sci. USA **109**, 3868–3873. (10.1073/pnas.1118372109) 22315416 PMC3309752

[9] Woodrow C, Celiker E, Montealegre-Z F. 2023 An Eocene insect could hear conspecific ultrasounds and bat echolocation. Curr. Biol. **33**, 5304–5315. (10.1016/j.cub.2023.10.040) 37963458

[10] Bailey NW, Pascoal S, Montealegre-Z F. 2019 Testing the role of trait reversal in evolutionary diversification using song loss in wild crickets. Proc. Natl Acad. Sci. USA **116**, 8941–8949. (10.1073/pnas.1818998116) 30992379 PMC6500131

[11] Pascoal S, Cezard T, Eik-Nes A, Gharbi K, Majewska J, Payne E, Ritchie MG, Zuk M, Bailey NW. 2014 Rapid convergent evolution in wild crickets. Curr. Biol. **24**, 1369–1374. (10.1016/j.cub.2014.04.053) 24881880

[12] Gallagher JH, Zonana DM, Broder ED, Herner BK, Tinghitella RM. 2022 Decoupling of sexual signals and their underlying morphology facilitates rapid phenotypic diversification. Evol. Lett. **6**, 474–489. (10.1002/evl3.302) 36579170 PMC9783451

[13] Bennet-Clark HC. 2003 Wing resonances in the Australian field cricket Teleogryllus oceanicus. J. Exp. Biol. **206**, 1479–1496. (10.1242/jeb.00281) 12654887

[14] Bailey NW, Zuk M, Tinghitella RM. 2024 Quiet but not forgotten: insights into adaptive evolution and behavior from 20 years of (mostly) silent Hawaiian crickets. In Advances in the study of behavior (eds J Podos, S Healy), pp. 51–87. Cambridge, MA: Academic Press. (10.1016/bs.asb.2024.03.001)

[15] Woodrow C, Baker E, Jonsson T, Montealegre-Z F. 2022 Reviving the sound of a 150-year-old insect: the bioacoustics of Prophalangopsis obscura (Ensifera: Hagloidea). PLoS One **17**, e0270498. (10.1371/journal.pone.0270498) 35947546 PMC9365155

[16] Mhatre N, Montealegre-Z F, Balakrishnan R, Robert D. 2012 Changing resonator geometry to boost sound power decouples size and song frequency in a small insect. Proc. Natl Acad. Sci. USA **109**, E1444–E1452. (10.1073/pnas.1200192109) 22547790 PMC3365161

[17] Godthi V, Pratap R. 2015 Dynamics of cricket sound production. J. Vib. Acoust **137**, 041019. (10.1115/1.4030090)

[18] Godthi V, Balakrishnan R, Pratap R. 2022 The mechanics of acoustic signal evolution in field crickets. J. Exp. Biol. **225**, jeb243374. (10.1242/jeb.243374) 35258611

[19] Bennet-Clark HC, Bailey WJ. 2002 Ticking of the clockwork cricket: the role of the escapement mechanism. J. Exp. Biol. **205**, 613–625. (10.1242/jeb.205.5.613) 11907051

[20] Jonsson T, Montealegre-Z F, Soulsbury CD, Robert D. 2021 Tenors not sopranos: bio-mechanical constraints on calling song frequencies in the Mediterranean field-cricket. Front. Ecol. Evol. **9**. (10.3389/fevo.2021.647786)

[21] Tinghitella RM, Broder ED, Gurule-Small GA, Hallagan CJ, Wilson JD. 2018 Purring crickets: the evolution of a novel sexual signal. Am. Nat. **192**, 773–782. (10.1086/700116) 30444653

[22] Schneider WT, Rutz C, Hedwig B, Bailey NW. 2018 Vestigial singing behaviour persists after the evolutionary loss of song in crickets. Biol. Lett. **14**, 20170654. (10.1098/rsbl.2017.0654) 29445043 PMC5830660

[23] Aberle B, Jemmali R, Dirks JH. 2017 Effect of sample treatment on biomechanical properties of insect cuticle. Arthropod Struct. Dev. **46**, 138–146. (10.1016/j.asd.2016.08.001) 27495946

[24] Klocke D, Schmitz H. 2011 Water as a major modulator of the mechanical properties of insect cuticle. Acta Biomater. **7**, 2935–2942. (10.1016/j.actbio.2011.04.004) 21515418

[25] Vincent JFV, Wegst UGK. 2004 Design and mechanical properties of insect cuticle. Arthropod Struct. Dev. **33**, 187–199. (10.1016/j.asd.2004.05.006) 18089034

[26] Dirks JH, Taylor D. 2012 Fracture toughness of locust cuticle. J. Exp. Biol. **215**, 1502–1508. (10.1242/jeb.068221) 22496286

[27] Wootton RJ. 1992 Functional morphology of insect wings. Annu. Rev. Entomol. **37**, 113–140. (10.1146/annurev.en.37.010192.000553)

[28] Salcedo MK, Socha JJ. 2020 Circulation in insect wings. Integr. Comp. Biol. **60**, 1208–1220. (10.1093/icb/icaa124) 32870980

[29] Pascoal S, Liu X, Ly T, Fang Y, Rockliffe N, Paterson S, Shirran SL, Botting CH, Bailey NW. 2016 Rapid evolution and gene expression: a rapidly evolving Mendelian trait that silences field crickets has widespread effects on mRNA and protein expression. J. Evol. Biol. **29**, 1234–1246. (10.1111/jeb.12865) 26999731

[30] Shankar S, Bryde P, Mahadevan L. 2022 Geometric control of topological dynamics in a singing saw. Proc. Natl Acad. Sci. USA **119**, e2117241119. (10.1073/pnas.2117241119) 35446615 PMC9169918

[31] ter Hofstede HM, Schöneich S, Robillard T, Hedwig B. 2015 Evolution of a communication system by sensory exploitation of startle behavior. Curr. Biol. **25**, 3245–3252. (10.1016/j.cub.2015.10.064) 26687622

[32] Souza-dias PGB, Szinwelski N, Fianco M, Oliveira ECD, Mello F, Zefa E. 2017 New species of Endecous (Grylloidea, Phalangopsidae, Luzarinae) from the Iguaçu National Park (Brazil), including bioacoustics, cytogenetic and distribution data. Zootaxa **4237**, 454–470. (10.11646/zootaxa.4237.3.2) 28264277

[33] Gorochov AV, Tan MK. 2012 New crickets of the subfamilies Phaloriinae and Pteroplistinae (Orthoptera: Gryllidae) from Singapore. Zootaxa **3525**, 18–34. (10.11646/zootaxa.3525.1.2)

[34] Tan MK, Kamaruddin KN. 2016 A new species of Gryllotalpa mole cricket (Orthoptera: Gryllotalpidae: Gryllotalpinae) from Peninsular Malaysia. Zootaxa **4066**, 552–560. (10.11646/zootaxa.4066.5.3) 27395853

[35] Tan MK, Japir R, Chung AYC, Wahab R. 2020 New taxa of crickets (Orthoptera: Grylloidea: Phaloriinae, Phalangopsinae, Itarinae and Podoscirtinae) from Borneo (Brunei Darussalam and Sandakan). Zootaxa **4810**, 244–270. (10.11646/zootaxa.4810.2.2) 33055895

[36] Jaiswara R, Dong J, Ma L, Yin H, Robillard T. 2019 Taxonomic revision of the genus Xenogryllus bolívar, 1890 (Orthoptera, Gryllidae, Eneopterinae, Xenogryllini). Zootaxa **4545**, 301–338. (10.11646/zootaxa.4545.3.1) 30790904

[37] Turchyn N, Popadic A. 2024 Role of nubbin in the development of forewing sound-producing structures in Acheta domesticus (house cricket). Front. Ecol. Evol. **12**. (10.3389/fevo.2024.1411228)

[38] Montealegre ZF, Ogden J, Jonsson T, Soulsbury CD. 2017 Morphological determinants of signal carrier frequency in katydids (Orthoptera): a comparative analysis using biophysical evidence of wing vibration. J. Evol. Biol. **30**, 2068–2078. (10.1111/jeb.13179) 28921699

[39] Chintauan‐Marquier IC, Legendre F, Hugel S, Robillard T, Grandcolas P, Nel A, Zuccon D, Desutter‐Grandcolas L. 2016 Laying the foundations of evolutionary and systematic studies in crickets (Insecta, Orthoptera): a multilocus phylogenetic analysis. Cladistics **32**, 54–81. (10.1111/cla.12114) 34732023

[40] Montealegre ZF, Windmill JFC, Morris GK, Robert D. 2009 Mechanical phase shifters for coherent acoustic radiation in the stridulating wings of crickets: the plectrum mechanism. J. Exp. Biol. **212**, 257–269. (10.1242/jeb.022731) 19112145

[41] Robillard T, Montealegre-Z F, Desutter-Grandcolas L, Grandcolas P, Robert D. 2013 Mechanisms of high frequency song generation in brachypterous crickets and the role of ghost frequencies. J. Exp. Biol. **216**, 2001–2011. (10.1242/jeb.083964) 23430987

[42] Thompson DW. 1992 On growth and form. Cambridge, UK: Cambridge University Press.

[43] Blankers T, Block R, Hennig RM. 2018 Codivergence but limited covariance of wing shape and calling song structure in field crickets (Gryllus). Evol. Biol. **45**, 144–155. (10.1007/s11692-017-9439-2)

[44] Pitchers WR, Klingenberg CP, Tregenza T, Hunt J, Dworkin I. 2014 The potential influence of morphology on the evolutionary divergence of an acoustic signal. J. Evol. Biol. **27**, 2163–2176. (10.1111/jeb.12471) 25223712 PMC4199928

[45] Weiner R, Duke S, Simonelli G *et al*. Reliable Reconstruction of Cricket Song from Biophysical Models and Preserved Specimens. Dryad (10.5061/dryad.v15dv4266) PMC12307059 40740698

[46] Weiner R, Duke S, Simonelli G, Bailey NW, Mhatre N. 2025 Supplementary material from: Reliable reconstruction of cricket song from biophysical models and preserved specimens. FigShare (10.6084/m9.figshare.c.7942440) PMC12307059 40740698

